# Hemoglobin Oxygen Affinity in Patients with Cystic Fibrosis

**DOI:** 10.1371/journal.pone.0097932

**Published:** 2014-06-11

**Authors:** Dieter Böning, Angela Littschwager, Matthias Hütler, Ralph Beneke, Doris Staab

**Affiliations:** 1 Institut für Sportmedizin, Charité - Universitätsmedizin Berlin, Berlin, Germany; 2 Klinik für Pädiatrische Pneumologie und Immunologie, Charité - Universitätsmedizin Berlin, Berlin, Germany; National Cancer Institute, United States of America

## Abstract

In patients with cystic fibrosis lung damages cause arterial hypoxia. As a typical compensatory reaction one might expect changes in oxygen affinity of hemoglobin. Therefore position (standard half saturation pressure P_50_st) and slope (Hill’s n) of the O_2_ dissociation curve as well as the Bohr coefficients (BC) for CO_2_ and lactic acid were determined in blood of 14 adult patients (8 males, 6 females) and 14 healthy controls (6 males, 8 females). While Hill’s n amounted to approximately 2.6 in all subjects, P_50_st was slightly increased by 1mmHg in both patient groups (controls male 26.7±0.2, controls female 27.0±0.1, patients male 27.7±0.5, patients female 28.0±0.3 mmHg; mean and standard error, overall p<0.01). Main cause was a rise of 1–2 µmol/g hemoglobin in erythrocytic 2,3-biphosphoglycerate concentration. One patient only, clearly identified as an outlier and with the mutation G551D, showed a reduction of both P_50_st (24.5 mmHg) and [2,3-biphosphoglycerate] (9.8 µmol/g hemoglobin). There were no differences in BCCO_2_, but small sex differences in the BC for lactic acid in the controls which were not detectable in the patients. Causes for the right shift of the O_2_ dissociation curve might be hypoxic stimulation of erythrocytic glycolysis and an increased red cell turnover both causing increased [2,3-biphosphoglycerate]. However, for situations with additional hypercapnia as observed in exercising patients a left shift seems to be a more favourable adaptation in cystic fibrosis. Additionally when in vivo PO_2_ values were corrected to the standard conditions they mostly lay left of the in vitro O_2_ dissociation curve in both patients and controls. This hints to unknown fugitive factors influencing oxygen affinity.

## Introduction

Cystic fibrosis (CF) is the most frequent genetic disease in Caucasians [1]–[3]. Mutations on chromosome 7 (location 7q31.2) reduce the effectiveness of the cystic fibrosis transmembrane conductance regulator (CFTR), which is essential for the secretion of chloride (Cl^−^) and consequently water in many glands. The clinical manifestation with heaviest impact is the progressive pulmonary disease. Because of the resulting deteriorated lung function in patients with cystic fibrosis causing hypoxia and partly also hypercapnia one might expect compensatory reactions in concentration and oxygen affinity of hemoglobin (Hb) to secure oxygen loading in spite of the reduced oxygen pressure (PO_2_) in pulmonary capillaries.

There are various strategies of defense against arterial hypoxia [4]–[8]. In addition to hyperventilation most healthy humans, except partly Tibetans and Ethiopeans [9]–[11], react to hypoxia with an increase in Hb concentration ([Hb]) which facilitates sufficient binding of oxygen at lowered PO_2_ in the lungs. Furthermore a right shift of the oxygen dissociation curve (ODC) under standard conditions (pH 7.4, PCO_2_ 40 mmHg, 37°C) partly compensates for the reduced diffusion pressure in the tissues because of the low oxygen saturation (SO_2_) in the capillaries; the shift is caused by more 2,3-biphosphoglyrate (BPG) in the red cells. In contrast typical altitude animals like llamas, guinea pigs and partly birds possess left-shifted ODCs securing oxygen loading in the lungs and rather low [Hb] reducing circulatory resistance. In addition small red blood cells and a dense capillary net in the tissues diminish the diffusion distance and thus compensate for the decreased capillary PO_2_ [12]. The human fetus exists also at very low arterial PO_2_ but the concentration of the high affinity fetal Hb (HbF) is increased. Recent in vivo determinations of the ODC in adults point to a possible left shift of its upper part at altitude [13], [14].

Astonishingly few studies investigated the combined effect of hypoxia and hypercapnia. The fetal conditions with higher arterial PCO_2_ than in maternal blood point to an advantage of a left shifted ODC. Moles living in earth holes with reduced air exchange inspire hypoxic/hypercapnic gas and possess also Hb with increased oxygen affinity [15]. Huckauf et al ([16]) describe a left shifted ODC in patients with chronic obstructive lung disease and in a review Morgan [17] mentions that [BPG] is often reduced in critically ill patients.

Patients with cystic fibrosis often show normal or even anemic [Hb] (e. g. [18]–[20]. Interestingly, however, they may possess an increased red cell volume masked by a concomitant rise in plasma volume [21], [22]. Compensatory reactions of oxygen affinity in cystic fibrosis have been investigated rarely. Slight right shifts of the standard ODC, characterized by a rise of P_50_st and caused by increased [BPG], were detected by some authors [18], [23], while others found unchanged [BPG] or P_50_st [19], [24].

However, there are various additional mechanisms for the regulation of oxygen affinity. Besides phosphates other anions like lactate, chloride and glutathione (e. g. [25], [26]) bind to Hb. Depending on the binding site these substances also influence the cooperativity of the subunits visible as change in the slope of the ODC (Hill’s n); additionally they may modify the intraerythrocytic pH. The Bohr effect, which in the physiological pH range causes an increase of PO_2_ at constant saturation by acidification (essential in working muscles), may vary depending on various factors like oxygen saturation [27], [28], type of acid [27], [28], substance concentrations and age of the erythrocytes (e. g. [29]); in altitude residents a tendency to lowered Bohr coefficients (BC = ΔlogPO_2_/ΔpH) has been observed [30], [31]. Also sex differences in oxygen binding properties have been described: women [32]–[34] as well as children [35] tend to higher P_50_st than men. Finally in vivo variations of the ODC in venous blood of anemic patients as well as of trained subjects have been observed during exercise which were no more detectable after in vitro equilibration of blood [36]–[38]. The underlying mechanisms are not yet clarified.

Previous studies on oxygen affinity in cystic fibrosis were performed on rather heterogeneous groups of patients. Differences in the severity of the illness are almost inevitable but possible effects of age and sex were not considered. Also control groups were small or not clearly defined or even lacking. To our knowledge neither cooperativity (Hill’s n) nor the Bohr effect have ever been studied in cystic fibrosis.

Considering all these factors it seemed worthwhile to perform a systematic study of mechanisms influencing blood O_2_ affinity as possible facilitation of oxygen uptake in cystic fibrosis.

## Methods

### Study Participants

Measurements were performed in 14 adult patients and 14 controls; anthropometric data are presented in Table 1. The patients (8 males, 6 females) showed severely reduced lung function but were in a stable clinical condition. One male subject was bearer of the Class III G551D mutation which is one of five mutations with a frequency >0.1% accounting for 2 to 3% mutations worldwide. It impairs CFTR-mediated Cl^−^ transport by limiting channel gating at the cell surface [3], [39].

**Table 1:** Subjects.

		Age	Body mass	Height	BMI	FVC	FEV1	PEF
	n	years	kg	cm	kg/m^2^	%	%	%
Controls male	6	28±3	80.4±3.2	185±4	24.2±1.2	93±2	92±3	102±8
Controls female	8	27±2	62.8±3.0	171±3	21.5±0.8	117±4	104±5	98±10
Patients male	8	28±2	59.0±3.0	177±4	18.8±0.6	59±7	38±6	57±5
Patients female	6	29±1	48.8±1.7	162±2	18.6±0.5	56±8	37±6	51±12

Means and standard errors (SE). BMI body mass index, FVC forced vital capacity, FEV1 forced expiratory volume during 1 s; PEF peak expiratory flow. % of expected values for age and sex [40] or of individual FVC. All anthropometrical and lung function values are significantly (P<0.001) reduced in the patients.

The patients were the members of a group with exercise therapy. They usually lived at home but were under continuous supervision by physicians of the pediatric clinic of the faculty. Twice a week they performed a disease status tailored exercise program addressing endurance, strength, coordination and flexibility supervised by staff of the Institute of Sports Medicine and received individual advice for additional daily exercises at home. Occasionally some patients used short term oxygen supplementation, but not on the test day. The nonsmoking controls (6 males, 8 females) were physically active but not specifically or regularly training staff members and students. One female was slightly anemic ([Hb] 11.2 g/dl), but all other measurements yielded clinically normal values within the range of the group. The study protocol was approved by the ethics committee of the faculty (Ethikkommission, Charité – Universitätsmedizin Berlin, Ethikausschuss CBF, No. ek.185-13b) and written informed consent was obtained from all participants.

### Study Procedure

The subjects arrived at the laboratory between 9.00 and 10.00 a.m. Lung function (forced vital capacity FVC, forced expiratory volume during 1 s FEV1, peak expiratory flow PEF) was measured with a spirometer system (Oxycon gamma, Mijnhardt, Bunnik, The Netherlands). Percent of expected values for age and sex [40] or of individual FVC are presented in Table 1. Blood was sampled in supine position. Acid base status at 37°C (ABL 500 or 510 with no systematic difference between apparatus, Radiometer Copenhagen, Denmark), oxygenation status (PO_2_, SO_2_, COHb, MetHb) and [Hb] (OSM 3; Radiometer Copenhagen, Denmark) were measured in heparinized blood samples taken from hyperemized ear-lobes. Values for PO_2_ are slightly lower than in arterial blood [41], but this is of negligible importance for saturations above 90% in the flat part of the ODC. Fifty ml of venous blood were drawn without stasis using heparinized vaccutainers and stored in an ice-water mixture. Oxygenation status, [Hb], hematocrit (Hct, microhematocrit method) and [Cl^−^] in plasma (EML 100, Radiometer Copenhagen) were determined immediately. Aliquots were deproteinized and stored at −20°C for duplicate measurements of ATP and BPG concentrations (enzymatic kits, Sigma Diagnostics) on the next day.

Five ml each were equilibrated 20 min in sphere tonometers at 37°C with air/CO_2_ or nitrogen/CO_2_ mixtures (3, 6 or 10% CO_2_). Lactic acid (13.5 mmol/l blood) was added to an additional sample equilibrated thereafter with 6% CO_2_ in air or N_2._ After taking aliquots for additional ATP and BPG measurements 0.2 ml of oxygenated blood were successively added 8 to 10 times to 1 ml deoxygenated blood using 2 connected syringes and mixed. After measurement of SO_2_, COHb, MetHb, [Hb], pH, PCO_2_ and PO_2_, ODCs were drawn in the Hill plot (log SO_2_/100-SO_2_) versus log PO_2_).

Samples of native blood as well as of blood equilibrated with N_2_/6% CO_2_ and with air/6% CO_2_ were centrifuged for 10 min (3500 rpm, 4°C). Part of the red cell sediment was hemolyzed by repeated freezing and thawing and used for measurement of pH and [Cl^−^] in the erythrocytes.

Twelve patients (7 males, 5 females) performed an incremental test (initially 0.3 W/kg, plus 0.3 W/kg every 2 min) until exhaustion on a cycle ergometer (Lode Excalibur, The Netherlands) during exercise therapy. Blood gases and lactate concentration (Ebio plus, Eppendorf, Germany) were measured in ear lobe blood and used to calculate P_50_ at exhaustion.

### Calculations

The slope n of the oxygen dissociation curves linearized in the Hill plot served as measure of cooperativity. For 5% steps of SO_2_ between 15 and 90% logPO_2_ values were calculated from the regression equations and the corresponding pH values obtained by interpolation. Comparison of the ODCs for 3 and 10% CO_2_ yielded Bohr coefficients for CO_2_ (BCCO_2_), comparison of the 6% CO_2_ and the 13.5 mmol/l lactic acid curves yielded Bohr coefficients for fixed acid (BCLa) at each saturation step. P_50_st were calculated from the curves of blood equilibrated with 6% CO_2_ by use of the corresponding individual BCCO_2_. Mean cellular hemoglobin concentrations (MCHC) calculated from [Hb] and Hct were corrected for 2% trapped plasma. [Cl^−^]_ery_ were corrected for 10% in the sediment after centrifugation with 3500 rpm; because of the large buffer capacity of red cells this is not necessary for pH_ery_. Electrodes in the electrolyte analyser measure concentrations in water [42]; therefore [Cl^−^]_ery_ is given per l cell water. Values for the control subjects coincide with titrimetric measurements [43]. In vitro blood buffer capacities (−Δ[acid]/ΔpH) for CO_2_ and lactic acid were calculated from the measurements in the corresponding equilibrated samples.

### Statistics

All data are presented as means±standard errors (SE). Dependent on the number of comparisons, t-tests or analysis of variance (ANOVA) were used for significance calculations. The probability that an outlier does not belong to a sample was tested eventually [44]. Differences with P<0.05 were considered as significant.

## Results

### Anthropometry and Pathology

Table 1 shows marked reduction in both body height and body mass in the patients compared to healthy subjects. Their lung function was substantially impeded by restrictive as well as obstructive damage visible from low vital capacity and expiratory flow (FEV1, PEV); FEV1 ranged between 22 and 74%.

### Blood Gases and Acid Base Status

The impaired lung function of the patients caused a reduction of ear-lobe PO_2_ and SO_2_ (Table 2). Generally these values were also slightly lower in males than in females. Correspondingly, PCO_2_ tended to higher values in males and in patients. However, the pH was equal in all subgroups because of non-respiratory compensation visible as increased base excess in males and in patients. Venous blood pH scattered more, but there were also no systematic differences among groups (means between 7.35 and 7.38); red cell pH showed no influence of sex or illness as well (means about 7.16). In vitro buffer capacities of blood tended to higher values in all males; this was significant for acidification with CO_2_ as well as lactic acid (both P<0.05; latter not shown in Table 2) in oxygenated blood. Concentrations of COHb (controls male 0.6±0.1%, controls female 0.4±0.1%, patients male 0.6±0.2%, patients female 0.7±0.1%) and MetHb (controls male 0.5±0.1%, controls female 0.6±0.1%, patients male 0.5±0.1%, patients female 0.5±0.1%) were low and not different among groups.

**Table 2:** Blood gases and acid-base status.

		PO_2art_	SO_2art_	PCO_2art_	pH_art_	SBE	Buffer Cap
	n	mmHg	%	mmHg		mmol/l	mmol/l
Controls male	6	91.4±3.0	95.9±0.3	38.0±1.6	7.430±0.006	1.3±0.7	29.9±1.2
Controlsfemale	8	99.3±1.2	96.8±0.2	34.4±1.6	7.430±0.008	−0.9±0.7	25.2±1.0
Patients male	8	64.9±3.5	91.2±1.5	41.7±1.8	7.423±0.008	3.4±0.5	28.9±0.7
Patients female	6	70.5±2.7	93.3±0.7	37.2±2.0	7.427±0.012	1.4±0.7	26.8±1.0
Anova	sex	a		a		b	a
	illness	c	d			d	

Means ± SE. art measurements in ear lobe blood. Standard base excess (SBE, 100% SO_2_, standardized [Hb] of 5 g/dl, [71]) measured in venous blood. Buffer Cap: in vitro buffer capacity for CO_2_ in oxygenated blood (-Δ[HCO_3_^−^]_plasma_/Δ pH). ANOVA: a P<0.05, b P<0.01 or better for differences between males and females, c P<0.05, d P<0.01 or better for differences between controls and patients.

### Blood Composition

[Hb] and Hct were higher in males than females, but there were no significant differences between controls and patients (Table 3). In the male patients [Hb] was negatively correlated with FEV1 (r = −0.762, P<0.05). MCHC, however, was slightly but significantly lowered in patients. They showed also slightly decreased [Cl^−^] in plasma and red cells. [BPG] and [ATP] were significantly increased in the patients inspite of very low [BPG] (9.8 µmol/gHb) in the subject with the G551D mutation. Additionally there was a sex difference for [BPG] with higher concentrations in females. After equilibration [BPG] and [ATP] tended to slightly higher values (0.9 and 0.2 µmol/gHb on average, respectively, not significant) compared to native blood.

**Table 3:** Substance concentrations in venous blood.

		[Hb]	Hct	MCHC	[Cl^−^]_plasma_	[Cl^−^]_ery_	[BPG]_ery_	[ATP]_ery_
	n	g/dl	%	g/dl	mmol/l	mmol/l H_2_O	µmol/gHb	µmol/gHb
Controls male	6	15.4±0.4	45.7±0.8	33.8±0.5	103.3±0.5	73.9±1.1	13.3±1.2	4.1±0.3
Controls female	8	12.8±0.4	39.6±0.2	32.8±0.3	103.2±0.4	76.0±1.5	14.6±0.8	4.2±0.2
Patients male	8	14.7±0.4	46.5±1.5	31.9±0.4	99.6±0.5	71.9±1.9	14.1±0.8*	4.6±0.1
Patients female	6	12.9±0.4	42.0±1.3	31.8±0.6	100.3±1.2	73.0±1.3	16.7±1.5	4.3±0.3
Anova	sex	a	a				a	
	illness			c	d	c	c	d

Means ± SE. Significance levels indicated like for Table 2. MCHC and [Cl^−^]_ery_ corrected for 2% and 10% trapped plasma, respectively. *14.8±0.5 without the patient with the G551D mutation.

### Oxygen Dissociation Curves

**Figure 1:**
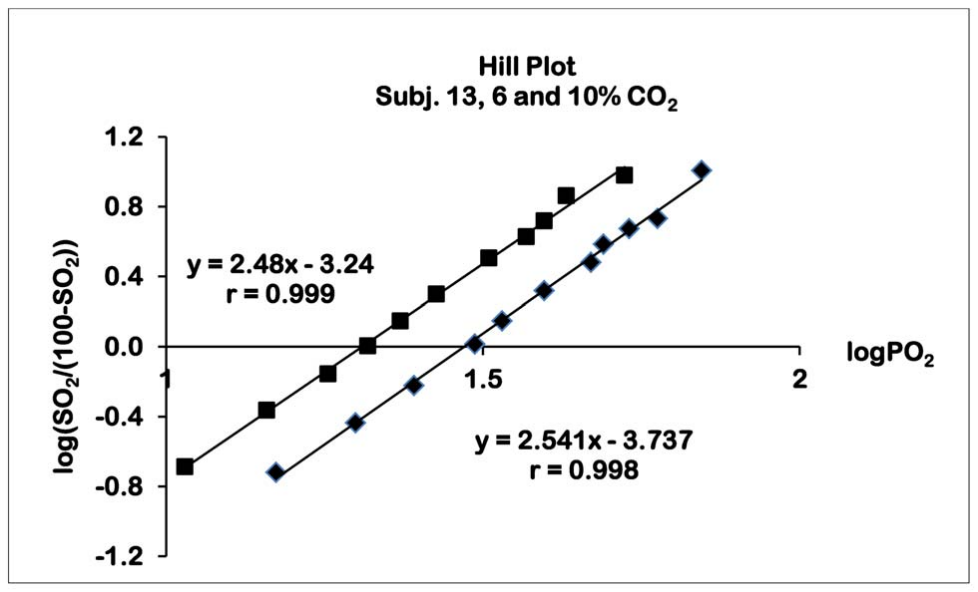
Two oxygen dissociation curves of one subject in the Hill plot.

In the Hill plot (Fig. 1) all curves were linear (correlation coefficients better than 0.98, not corrected for the slightly decreasing pH with rising saturation) and the slopes amounted to approximately 2.6 with very little scattering in all groups (Table 4).

**Table 4:** Characteristics of the oxygen dissociation curves.

		P_50_standard	Hill’s n
	n	mmHg	6% CO_2_
Controls male	6	26.7±0.2	2.63±0.02
Controls female	8	27.0±0.1	2.56±0.02
Patients male	8	27.7±0.5*	2.57±0.03
Patients female	6	28.0±0.3	2.61±0.03
Anova	sex		
	illness	d	

Means ± SE. Significance levels indicated as for Table 2. P_50_standard: standard half saturation pressure calculated from the curves of blood equilibrated with 6% CO_2._*28.1±0.3 mmHg without the patient with the G551D mutation.

The standard half saturation pressures (Table 4) corresponded to known normal values in the controls. In patients P_50_st was significantly increased by 1 mmHg (with slightly but not significantly higher values for females). When corrected to arterialized pH and PCO_2,_ all means were 0.8 mmHg lower. The patient with the G551D mutation presented a markedly lowered P_50_st of 24.5 mmHg (arterialized blood 23.3 mmHg) clearly identified as an outlier (P<0.02). Without the latter value mean P_50_st for the male patients rose by 0.4 mmHg.

**Figure 2:**
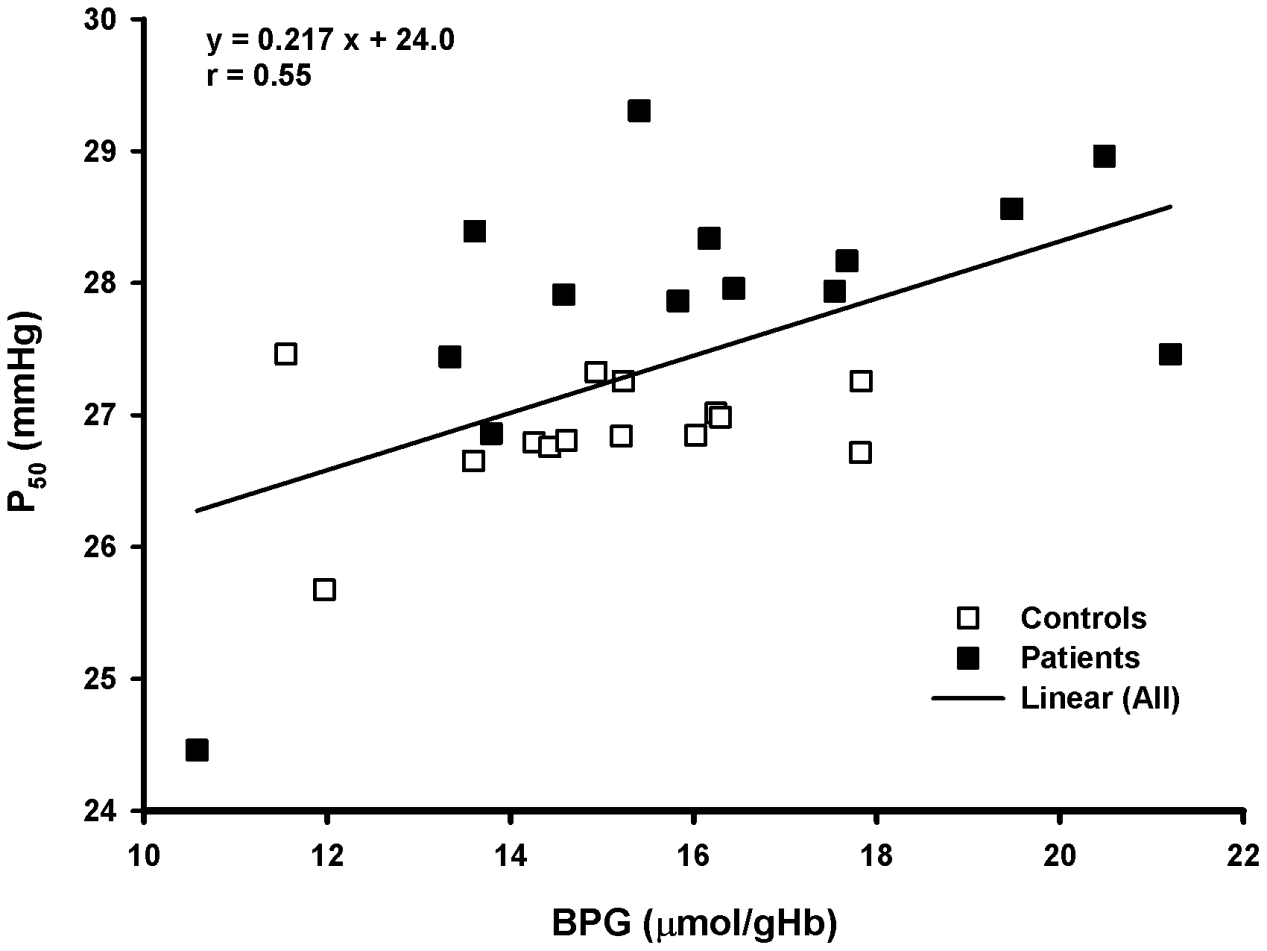
Dependence of P_50_st on BPG concentration (means of equilibrated samples). Regression line for all values, correlation coefficient r different from zero (P<0.01).

Regression analysis including all subjects yielded a significant relation between [BPG] in the equilibrated samples and P_50_st (Fig. 2). The male patient with the extremely low P_50_st value fell, however, far outside of his group with a correspondingly low [BPG].

### Bohr Coefficients

**Figure 3:**
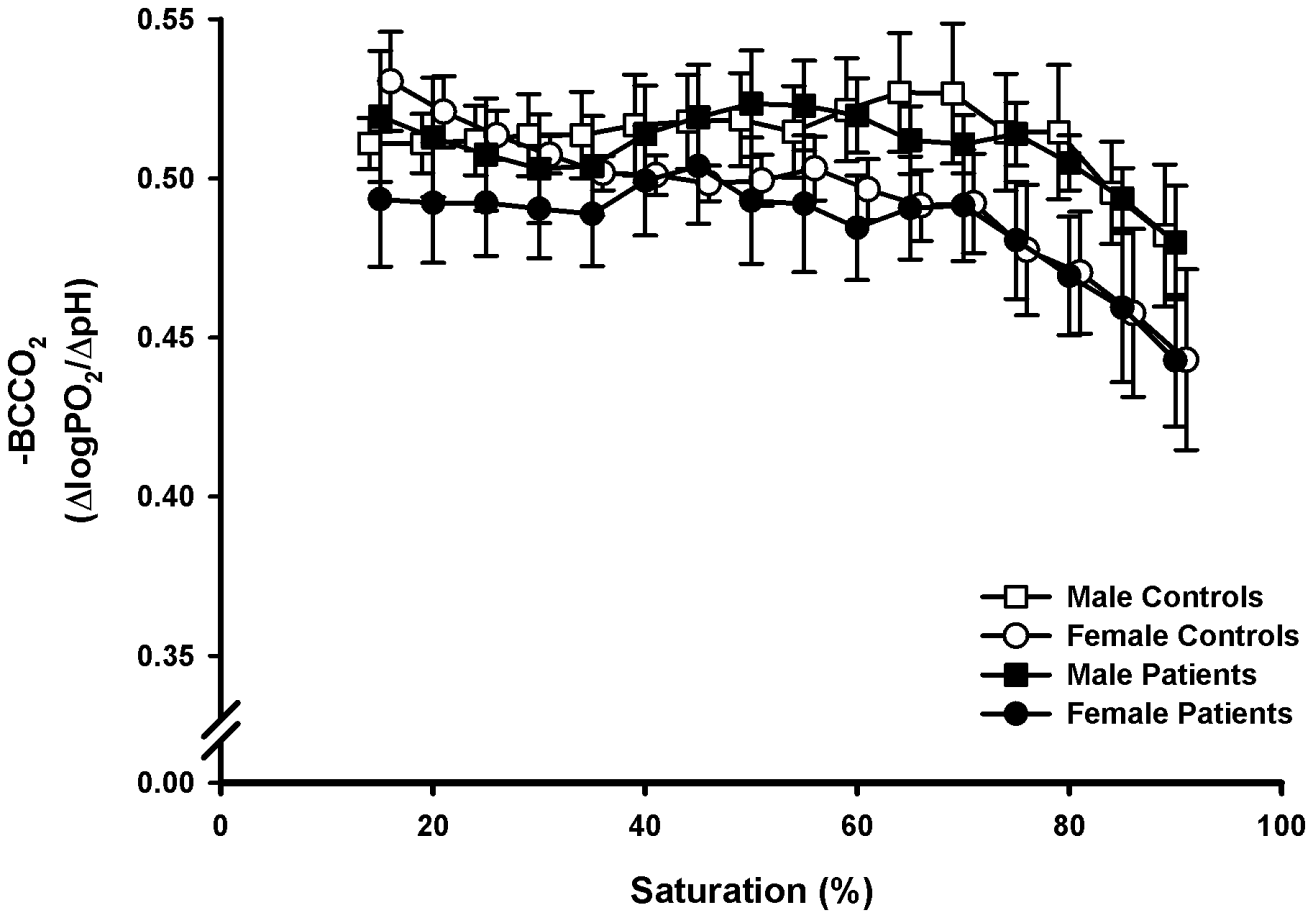
Saturation-dependent Bohr coefficients for acidification with CO_2_ (BCCO_2_). Means and standard errors.

**Figure 4:**
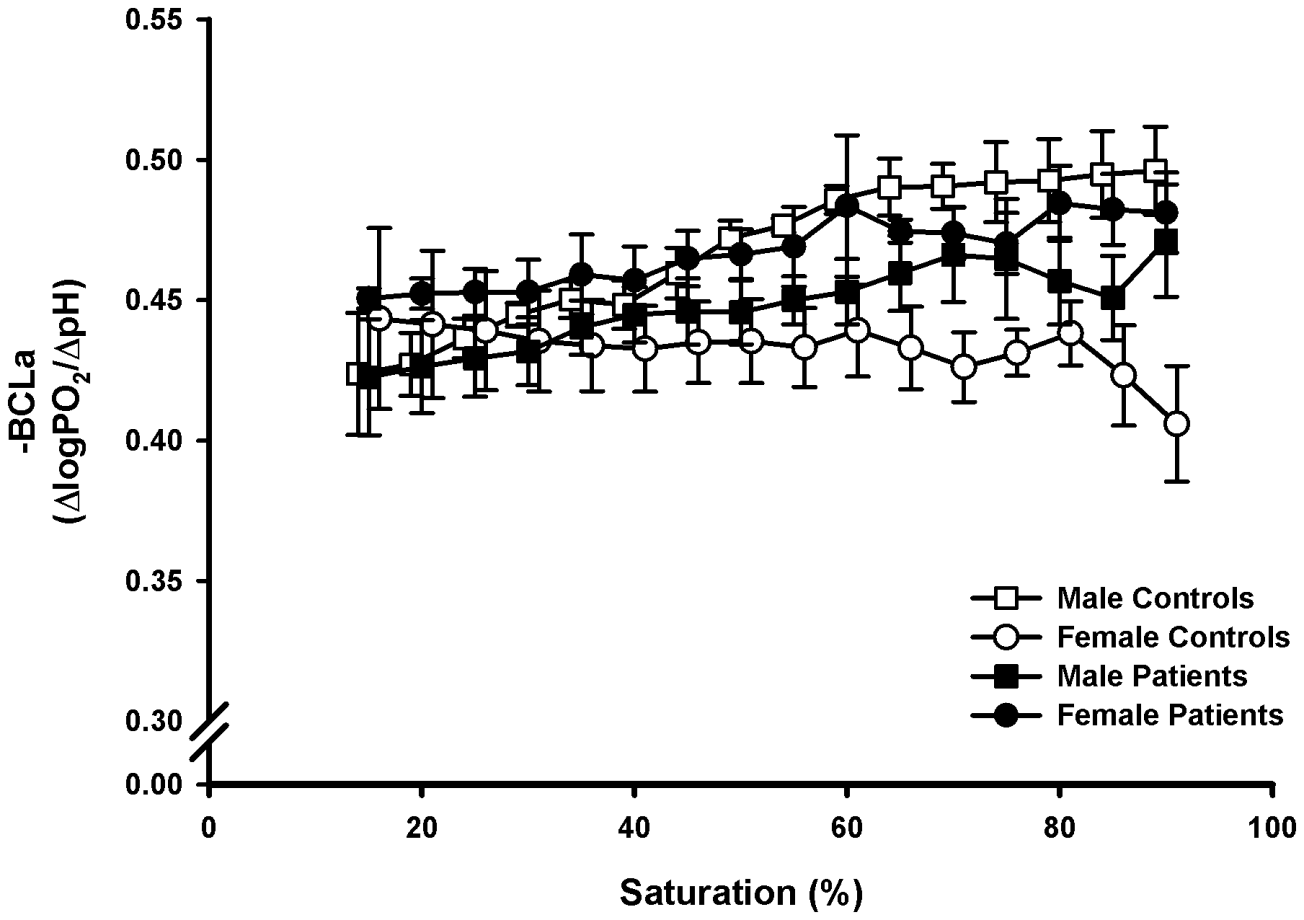
Saturation-dependent Bohr coefficients for acidification with lactic acid (BCLa). Means and standard errors.

The Bohr coefficients (Fig. 3) for CO_2_ corresponded to published data: The value was about −0.5 and decreased numerically with higher saturations (P<0.01 for all subjects); there was also a tendency to lower values in women. No influence of the disease was visible. The Bohr coefficients for lactic acid (Fig. 4) were generally lower numerically than for CO_2_ in all groups up to 45% saturation (−0.40 to −0.45). Differences between males and females at higher SO_2_ disappeared in the patients (interaction sex-illness P<0.01). Among the patients the subject with the G551D mutation presented the highest BC for both acids between 70 and 90% SO_2_ (approx. −0.54).

### Exercise Tests in Patients

At exhaustion SO_2_ dropped in all patients resulting from reduced PO_2_ and both respiratory and non-respiratory acidosis which caused a rise of P_50_ (Table 5). Again the subject with the G551D mutation showed the lowest P_50_ value (29.6 mmHg).

**Table 5:** Blood gases, acid-base status and P_50_ of patients at maximal exercise.

		Powermax	PO_2art_	SO_2art_	PCO_2art_	pH_art_	[Lactate]	P_50_
	n	Watt/kg	mmHg	%	mmHg		mmol/l	mmHg
Patients male	7	2.2±0.3	58.9±5.4	80.1±3.1	48.3±4.0	7.240±0.018	7.9±1.1	33.1±0.9
Patients female	5	1.8±0.1	55.6±2.1	82.6±4.0	51.2±3.6	7.227±0.031	8.8±0.6	33.7±0.9

Means ± SE. Measurements in ear lobe blood. P_50_ for actual pH. All differences to rest significant for all patients as well as subgroups except SO_2_ for subgroup patients female (P<0.054).

### In vivo Effects

**Figure 5:**
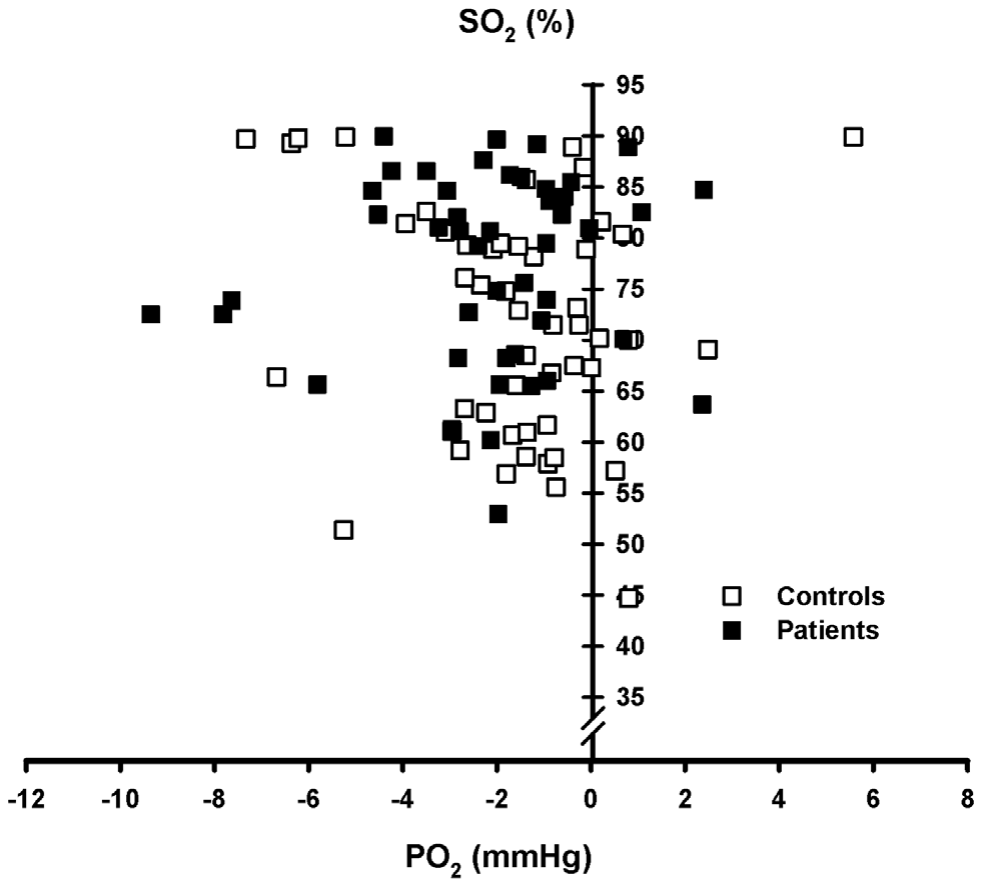
Deviation of in_2_ corrected to pH 7.4 from the corresponding individual standard ODC between 40 and 90% in controls and patients.

When the PO_2_ values in non-equilibrated venous blood (fresh or stored in ice until measurement) were corrected with the corresponding Bohr coefficients (BCCO_2_) to pH 7.4, they should have fallen on the individual standard ODC. However, in the range between 45 and 90% SO_2_ there was a tendency for a deviation to the left (Fig. 5) in controls (−1.8±0.4 mmHg, P<0.05) as well as in patients (−2.2±0.4 mmHg, P<0.001). Some samples with higher values of SO_2_ were not considered, because the BCs were not measured for SO_2_>90%. In addition there is large scattering of PO_2_ in the flat part of the ODC. There was no correlation between PO_2_ differences and [BPG] differences for native and equilibrated blood.

## Discussion

### Synopsis of Results

Our results confirm former investigations that there is a small right shift of the standard ODC in most patients with cystic fibrosis probably caused by slightly increased intraerythrocytic concentrations of organic phosphates [18], [23]. This is accompanied by a constant slope of the ODC and only small changes of the Bohr coefficients. In spite of the lacking hypocapnia this reaction is similar to the typical human acclimatization to altitude but seems to be attenuated. During exercise the right shift of the ODC is enforced by hypercapnia in CF patients and a clear drawback for arterial oxygen loading. Interestingly there seems to exist an additional mechanism in controls as well as in patients: The in vivo standard ODC falls slightly left of the in vitro curve.

### Blood Gases and Acid Base Status

The deterioration of lung function in the patients results in hypoxia visible in arterialized blood; a tendency to a slightly higher PCO_2_ than in the controls is not significant probably because of the low number of measurements and the resting situation; when exercising the increase in PCO_2_ is more marked. Measurements in 69 patients in our laboratory showed corresponding results; PaCO_2_ increased with the severity of the illness at rest as well as during exercise [45]. This is different to healthy subjects who always show a decrease of PaCO_2_ at high work load. The arterial oxygen saturation in patients at rest is as low as in highlanders [46], [47] living 2600 m above sea level (inspiratory PO_2_ approx. 120 mmHg). But in spite of a similar reduction of spirometric values the female patients show higher PO_2_ and lower PCO_2_ than the male patients like their healthy counterparts. Probably the long-known stimulation of respiratory brain centres by female hormones important for fetal oxygen supply is the cause (reviewed in [47]). The fact that arterialized pH is equal in all subgroups in spite of differences in PCO_2_ demonstrates the importance of acid-base homeostasis for physiological functions. Non-respiratory compensation is mainly done by renal excretion/reabsorption of bicarbonate. In the patients the osmotic effect of the rise of [HCO_3_^−^] is counteracted by a decrease of [Cl^−^]. Also the loss of chloride via sweat glands might play a role. The slightly increased in vitro buffer capacity in both male groups is obviously caused by the higher Hb concentration. In cystic fibrosis the slight rise in bicarbonate concentration as well as the possibly increased Hb mass [21], [22] help to attenuate the extracellular pH changes during exercise [48] caused by CO_2_ retention.

### Blood Composition

Hb concentrations showed typical sex differences but no sign of anemia in the patients. The latter might be expected in CF because of frequent problems with iron resorption. However, in our patients iron metabolism was routinely checked and deficiency was treated. One explanation for normal [Hb] in other studies might be the counteracting effects between iron deficiency and hypoxia [49]. Christoforou et al. [19] described a negative correlation of [erythropoietin] with FVC and FEV1. Such a dependency is probably the cause of the correlation between [Hb] and FEV1 in our male patients. Some authors [21], [22] have even observed an increase in red cell volume in CF probably stimulated by erythropoietin which might be explained as a typical hypoxia reaction. However, only in a fraction of the corresponding studies [19], [20], [50], [51] http://www.ncbi.nlm.nih.gov/ pubmed?term = McColley%20SA%5BAuthor%5D&cauthor = true&cauthor_uid = 21365780 erythropoietin concentration was increased. Own unpublished measurements support the idea of chronic stimulation of erythropoesis in CF patients based on elevated erythropoietin as well as soluble transferrin receptor concentrations in a cohort of 79 CF patients. Also a low MCHC like in the patients is often related to an increased water content typical for young erythrocytes. Furthermore in patients the high level of the soluble transferrin receptor [20] might be indicative for an increased red cell production and thus a reduced erythrocytic age. However, also a link between CFTR and the function of the hypoxia inducible factor has been put forward [52] which may serve as one potential reason for a lack of increased [Hb] in CF patients.

Factors possibly increasing [BPG] and [ATP] are low SO_2_ (reducing product inhibition because of BPG binding to Hb) and alkalosis (stimulating glycolysis and thus BPG synthesis). A probable explanation for the rather small increase of [BPG] and P_50_st in CF compared to highlanders with similarly lowered arterial SO_2_ and equal pH at rest (e. g. 18 µmol/g Hb in [30]) might be the different effect of physical activity: CO_2_ retention causes respiratory acidosis already during moderate physical activity in the patients while highlanders effectively hyperventilate at each exercise level. In the present patient group with normal daily life and exercise therapy physical activity was obviously a factor of some importance. Additionally a low red cell age as suggested above might lead to elevated [BPG] as well as [ATP] because of high enzymatic activity [29]. The low [BPG] in the patient with the G551D mutation possibly results from changed enzyme activities because no differences in erythrocyte physiology were detectable. CFTR is incorporated into the red cell membrane (e. g. [53]), but a relation to BPG metabolism remains speculative.

[Cl^−^] in red cells in part follows passively changes in plasma [Cl^−^] and therefore is lowered in patients. Generally the marked concentration difference results from the high erythrocytic content of non-diffusible anions (Hb^−^ and organic phosphates) causing a Donnan equilibrium. Cl^−^ crosses the cell membrane mainly through band 3 channels. The reduction of the number of CFTR molecules in patients (e. g. [53]) does not affect this exchange [24]. Cl^−^ concurs with BPG for the same binding sites on Hb [54] but its affinity is lower and the small decrease of its concentration in CF is compensated for by increased [BPG].

### Oxygen Dissociation Curves

The P_50_st values of controls scatter around the normal mean value (approximately 27 mmHg) without significant sex differences. The generally higher P_50_st in patients results from the increased [BPG] (change approx. 0.6 mmHg per µmole BPG/gHb according to [55]) while ATP plays only a minor role because of complexing with Mg^++^. This corresponds to the typical chronic hypoxic reaction of most humans. It allows to extract more oxygen in the tissue capillaries without lowering the diffusion pressure, but it is not helpful for oxygen loading in pulmonary capillaries. In highlanders with similar reduction of arterial SO_2_ P_50_st scatters around 30 mmHg [30]. In both healthy subjects and patients the reaction (affinity change, increased ventilation and partly stimulated erythropoesis) is a sufficient compensation of moderate hypoxia at rest but maximal performance capacity is reduced. The left shift of the ODC in moles [15] living and working under comparable conditions (inspiring air with reduced O_2_ and increased CO_2_ content) as the patients is more reasonable but is rare in humans. Under extreme acute conditions (above 6000 m of altitude) healthy mountaineers lower their P_50_ by extreme hyperventilation [56] which is not a sustainable option for CF-patients. For chronic acclimatization a reduction of [BPG] would be more appropriate. Surprisingly the male patient with the G551D mutation showed such an effect. One can estimate that a reduction of P_50_st like in his blood would raise the arterial SO_2_ at exhaustion in the male patients by 4%.

Interestingly in the 3 papers with P_50_st measurements in CF patients single low P_50_st values between 23.5 and 25.5 mmHg can be found [18], [19], [23]. This points to a special form of hypoxia acclimatization in some patients similar to that in moles.

The magnitude of a change in P_50_st may reflect further compensating mechanisms. Rosenthal et al [18] showed that P_50_st is negatively correlated with systemic oxygen delivery which depends on arterial SO_2_, [Hb] and cardiac output. This means that low [Hb] or cardiac output favor a rise of P_50_st. Indeed Arturson [57] described a P_50_st increase with falling [Hb] in chronic pulmonary insufficiency. The present CF patients were not anemic. This might also explain why we observed a small tendency rather than a substantial change in P_50_st.

Hill’s n did not deviate much from the usually expected value of 2.7 for HbA (e.g. [55]). BPG binds to Deoxy-Hb only which may therefore increase Hill’s n with rising concentration. A sligtly higher n in highlanders [30] and anemic patients [58] might be explained by this mechanism. However, the [BPG] differences between controls and patients in this study are too small to cause measurable effects.

### Bohr Effect and Exercise

Similar like in altitude inhabitants [30], [31] the Bohr coefficients are little changed in patients with cystic fibrosis. The coefficients for CO_2_ correspond to published values [27], [28], [33]. They are large (numerically) at low saturation because of oxygenation dependent binding of carbamate in addition to H^+^ effects during acidification with CO_2_. They are lower at very high saturation; the Bohr effect disappears when all Hb molecules are in the R (relaxed) state. Anions (Cl^−^, La^−^ and BPG) compete with CO_2_ at the terminal valines (e. g. [25]). Therefore BCCO_2_ increases at low [BPG]; this might be the cause for the rather high value in the patient with mutation G 551D. The fixed acid Bohr coefficients are small especially at low saturation compared to BCCO_2_. The slight unexplained influence of CF on BCLa plays only a very modest role, because the peak lactic acid concentration during exercise is rather low in the patients (Table 5) compared to healthy subjects (e. g. [59]). In the lungs the Bohr effect of CO_2_ is helpful for oxygen loading, when CO_2_ leaves the blood, especially during hyperventilation with resulting hypocapnia during heavy exercise. In the patients with exercise hypercapnia and mostly high P_50_st, however, an increase of BCCO_2_ would be detrimental in this situation.

### In vivo Effects

The left shift of the in vivo PO_2_/SO_2_ pairs relative to the in vitro standard ODCs is on an average modest (approx. 2 mmHg) but 17 differences amount to more than 4 mmHg. Differences in ODCs as well as BCs between fresh blood immediately after sampling and blood after equilibration in tonometers have occasionally been observed (e. g. [38], [60]). Concentration changes of BPG, ATP, Cl^−^, nitrocompounds or glutathione are possible causes. The means of [BPG] and [ATP] increase slightly but not significantly after equilibration compared to fresh venous samples explaining only 0.7 mmHg of the difference at 50% SO_2_. Intraerythrocytic [Cl^−^] changes are larger for a given ΔpH in vivo than in vitro resulting from exchange with the interstitial fluid [59]. Because of the opposite effects of SNO-Hb and Hb[FENO] on oxygen affinity [61] NO usually exerts no measurable influence on the ODC neither in vitro nor in vivo if no methemoglobin is formed [62]–[65]. In our experiments MetHb was stable. For an allosteric effect intraerythrocytic [NO] is by far too low even in Tibetans who present very high values [66]. In contrast to NO glutathione is present in millimolar concentrations in the red cells [67] and binds to oxy-Hb thus shifting the curve to the left and reducing the Bohr effect [26], [68]. But a marked deficiency of extra- and intracellular glutathione possibly including erythrocytes in CF patients has been suggested [69] which might be related to the disturbed function of CFTR as glutathione transporter [52]. Very recently also an effect of glutamate on P50 was observed [70]; this substance binds to Ca^++^ channel proteins in the cell membrane and may be interchanged with muscle fibres. Thus at the moment a clear cause for the in vivo – in vitro difference of PO_2_ remains unknown, but apparently it rises with SO_2_. This produces a left shift of the in vivo ODC between 50 and 90% SO_2,_ which is an advantage for oxygen loading. Recently similar results were found at 3600 m of altitude [13], [14]. Interestingly at low saturations “standardized” in vivo PO_2_/SO_2_ pairs tend to lie right of the in vitro curve, especially in venous blood returning from exercising muscles [36]–[38], [55], [60]. The result of this opposite changes is a markedly steepened complete in vivo oxygen dissociation curve probably in healthy subjects as well as in CF patients. Such a property is favorable for both loading and unloading of O_2_ in lungs and consuming tissues.

### Conclusions

The majority of the patients with cystic fibrosis in our study react to the problem of pulmonary oxygen uptake like man at altitude with a small right shift of the in vitro ODC caused by increased organic phosphate concentrations in the red cells. This improves oxygen diffusion into the consuming tissues, but is a drawback for arterialization. Healthy subjects can compensate this by hyperventilation thus reducing arterial PCO_2_ with resulting left shift of the ODC during oxygenation. This is not possible for CF patients especially when CO_2_ production is increased during exercise. A probably more appropriate left shift by reduction of [BPG] was observed in one patient with the G551D mutation. Also in other papers occasional left shifts can be detected. Whether this is a genetic effect, remains an intriguing question. The slope of the in vitro ODC and the Bohr coefficients were not markedly affected by the disease. Under in vivo conditions, there is a tendency for a left shift of the upper part of the ODC in both healthy controls and patients pointing to unknown affinity modifying factors which improve oxygen loading in the lungs.
